# 

**Published:** 1921-08-20

**Authors:** 


August 20 1921] [Price Twopence

### The Hospital

The Workers' Newspaper of Administrative Medicine and Institutional Life, Administration, National Insurance and Health.

CONTENTS

**Planning the Autumn Campaign** ... ... ... 341
**"Mass" Contributions in London** ... ... ... 342
Suggested Methods of Development.
**Notes of the Week** ... ... ... ... 343
Grants from the Voluntary Hospitals Commission—The "Sussex" Scheme—Higher Charges for Distant Patients?—The Lowestoft Experiment—A New Model Institution—Tragic Death of Dr. Cuff—The Russian Famine—Ancoats Hospital—Infirmary Patients and Medical Attention—Gifts in Kind for a Nurses' Home?—Antidotes to Poisons.
**The Smallpox Question** ... ... ... ... 345
Our Unvaccinated Population Steadily Increasing.
**The Problem of the Insane** ... ... ... ... 346
II. Condition of the Patients.
**Converting Coal-Burning Boiler Furnaces to Oil** 347
**An Encyclical from Charing Cross** ... ... 348
**The Finances of Metropolitan Hospitals** ... ... 348
**The Public Well-Being** ... ... ... ... 349
Unconscious Malingering in Childhood.
**Under Ideal Conditions** ... ... ... ... 349
**Municipal Hospital and Clinical Benefits** ... 350
**Parliament and the Professions** ... ... ... 351
**Forthcoming Publications** ... ... ... ... 352
**Medical Appointments** ... ... ... ... ... 352
**The Liverpool Council of Voluntary Aid** ... ... 352
**The Matrons' and Sisters' Department** ... ... 353
Better Teaching in the Schools.
**Round the Hospitals** ... ... ... ... 353
**Ringworm Clinics** ... ... ... ... ... 356
**Matrons' and Sisters' Appointments** ... ... 356
**The Hospital Position at Wakefield** ... ... 357
**A Novel Gift and Others** ... ... ... ... 357
**"Progress" and the Hospital Pageant...** ... 357
**The College of Nursing (Limited by Guarantee)** 358
**Dental Nurses in New Zealand** ... ... ... 358

